# Fullerol C_60_(OH)_24_ Nanoparticles and Drought Impact on Wheat (*Triticum aestivum* L.) during Growth and Infection with *Aspergillus flavus*

**DOI:** 10.3390/jof7030236

**Published:** 2021-03-22

**Authors:** Tihomir Kovač, Tihana Marček, Bojan Šarkanj, Ivana Borišev, Maja Ižaković, Katarina Jukić, Ante Lončarić, Tamara Krska, Michael Sulyok, Rudolf Krska

**Affiliations:** 1 Department of Applied Chemistry and Ecology, Faculty of Food Technology, Josip Juraj Strossmayer University of Osijek, Franje Kuhača 20, 31000 Osijek, Croatia; tihana.marcek@ptfos.hr (T.M.); maja.izakovic@ptfos.hr (M.I.); ante.loncaric@ptfos.hr (A.L.); 2 Department of Agrobiotechnology (IFA-Tulln), Institute of Bioanalytics and Agro-Metabolomics, University of Natural Resources and Life Sciences Vienna (BOKU), Konrad Lorenzstr. 20, 3430 Tulln, Austria; bsarkanj@unin.hr (B.Š.); tamara.krska@ffoqsi.at (T.K.); michael.sulyok@boku.ac.at (M.S.); rudolf.krska@boku.ac.at (R.K.); 3 Department of Food Technology, University North, Trg dr. Žarka Dolinara 1, 48000 Koprivnica, Croatia; 4 Department of Chemistry, Faculty of Sciences, University of Novi Sad, Biochemistry and Environmental Protection, Trg Dositeja Obradovića 3, 21000 Novi Sad, Serbia; ivana.borisev@dh.uns.ac.rs; 5 BC Institute for Production and Field Crops, Dugoselska 7, Dugo Selo, 10370 Rugvica, Croatia; kjukic@bc-institut.hr; 6 Institute for Global Food Security, School of Biological Sciences, Queen’s University Belfast, University Road, Belfast, Northern Ireland BT7 1NN, UK

**Keywords:** fullerol C_60_(OH)_24_ nanoparticles, drought, wheat (*Triticum aestivum* L.), mycotoxins, *Aspergillus flavus* NRRL 3251

## Abstract

Fullerol C_60_(OH)_24_ nanoparticles (FNP)-wheat-*A. flavus* interaction outcome is more complicated in the presence of drought. This study sheds light on how the presence of FNP affects food and feed safety from the perspective of mycotoxin contamination. The study aims to determine the influence of FNP at environmentally plausible concentrations on wheat growth under drought stress and on the aggressiveness of *A. flavus* during wheat germination, as well as the influence of FNP on the secondary metabolite profile during the inappropriate wheat storage. The co-occurrence of drought and FNP inhibited germination and shoot growth, while an application of FNP alone had no negative effect on plant growth. Wheat pre-treated with FNP showed a concentration dependent resistance pattern to *A. flavus* aggressiveness. Nevertheless, using a LC-MS/MS based multi-mycotoxin method, six secondary fungal metabolites: 3-nitropropionic acid (<LOD −775.7336 ± 10.7752 ng mL^−1^), aflatoxin B1 (<LOD −6.78 ± 0.43 ng mL^−1^) and B2 (<LOD −0.07 ± 0.00 ng mL^−1^), aflatoxicol (<LOD −0.37 ± 0.16 ng mL^−1^), kojic acid (<LOD −1337.87 ± 189.04 ng mL^−1^), and O-methylsterigmatocystin (<LOD −0.17 ± 0.00 ng mL^−1^), were detected. FNP affected secondary metabolism of *A. flavus* during inappropriate wheat storage and increased the concentration of secondary metabolites in a concentration-dependent pattern (3-nitropropionic acid and kojic acid). In addition, aflatoxicol production was provoked in FNP treated samples.

## 1. Introduction

Fullerols C_60_(OH)_24_ are oxidized fullerene C_60_ derivatives that are soluble in water, a result of the passage of fullerene C_60_ through different environmentally relevant routes and photoexcitation in the presence of oxygen [1]. In the environment, fullerene C_60_ is present in nano-sized form (nC_60_) and its environmentally relevant daughter product fullerol C_60_(OH)_24_ nanoparticles (FNP) has a tendency to form aggregates in water, with a size ranking from 1 to 100 nm in diameter, and most of them take up 10 nm [2,3,4,5,6]. The nC_60_ and FNP are the result of natural processes in geological materials, but their presence in the environment could be the result of accidental and intentional release processes such as domestic propane and natural gas combustion, fuel-gas burning, ethylene flames, candle emissions, and coal and biomass burning. Furthermore, fullerene has been found in spark-ignited, diesel engine and jet-fuel emissions. Polycyclic aromatic hydrocarbons can transform into soot containing fullerenes during combustion [7]. Release of nC_60_ in aerosol has been detected in the Mediterranean Sea atmosphere, Arizona State, and Eastern China, but also in almost all other parts of the environment, such as wastewaters, surface waters, river sediments, and soils [7,8,9,10,11,12]. Due to their high solubility in the water, FNP can easily enter into plants which serve as a storage reservoir, thereby effecting yield and, even more entering the food and feed chain with unpredictable consequences [13]. Nanomaterials have the ability to interact with the environment although their reactivity is still poorly understood. Much effort has been put into understanding the mechanisms of FNP action during their interaction with mycotoxigenic fungi [2,3,4,5,6]. Former research raises questions about the impact of FNP on real substrates for foodborne fungi. Furthermore, climate change dramatically impede plant growth and development, but also alter the composition of mycotoxigenic fungal community structure [14,15,16,17,18].

Drought is an abiotic stress factor, occurring in high frequency as a consequence of global climate change and has resulted in prolonged hot dry summers combined with irregular rainfall periods [19,20]. In plants, drought causes a complex response on a physiological, molecular and metabolomics level. For one, drought induces the generation of reactive oxygen species (ROS), causing oxidative stress. These particles endanger the electron transport chain, the structure of chloroplast proteins and thylakoid membranes, and also cause the degradation of cell proteins, inhibition of enzymes, and phospholipid bilayer disintegration [21,22,23]. All of these changes negatively affect plant growth and development.

Wheat (*Triticum aestivum* L.) is one of the most important cereal daily used in human consumption. Germination is the most sensitive developmental period during which drought can occur, so investing efforts in the application of different strategies to develop the seed tolerance to drought are needed [24]. Although some nanoparticles are toxic for plants [25,26], there is a growing body of evidence, which highlights the positive side of using FNP on them. For instance, foliar application of FNP alleviated the oxidative damage of sugar beet (*Beta vulgaris* L.) under drought [27]. Furthermore, fullerol C_60_-OH nanoparticles promoted the growth of wheat (*Triticum aestivum* L.), roots, and increased the chlorophyll content in leaves [13]. In rapeseed (*Brassica napus* L.) exposed to 15% polyethylene glycol (PEG) treatment, fullerol C_60(_OH)_27_ increased the germination, dry weight, photosynthesis, accumulation of reactive oxygen species (ROS), and activity of antioxidant enzymes in leaves [28]. Another study showed that FNP presence in the nutrient medium can accelerate barley roots elongation [29].

An interesting observation regarding FNP-plant and mycotoxigenic fungi interaction was described by Avanasi et al. (2014) [30]. Authors reported that nC_60_ release into the environment will not be highly bioavailable for plants and will persist longer in the soil. If they are transformed up to FNP, there is the question regarding the impact on plant cells that are infected by mycotoxigenic fungi on the field during plant growth.

The aim of this study was to determine the effect of FNP in three different concentrations on wheat under drought by tracking the germination, shoot length, and biochemical parameters. Wheat was chosen because it is a widespread crop and a natural substrate susceptible to infection by foodborne mycotoxigenic fungi *Aspergillus flavus* [31,32]. The second setup was devoted to testing impact of FNP on *A. flavus* aggressiveness during wheat germination and FNP influence on secondary metabolite profile of fungi during the wheat storage under unappropriated storage conditions. To the best of our knowledge, similar findings have not been reported. Our hypothesis was that FNP will affect all observed parameters with a high dependence on the applied concentration.

## 2. Materials and Methods

**Chemicals**. Potato dextrose agar, malt extract agar and sucrose were obtained from Biolife (Italy). AFT standard mix (B1, G1, B2, G2) was purchased from BioPure (Tulln, Austria). Acetonitrile and methanol (HPLC grade both) were obtained from Merck (Darmstadt, Germany). Ammonium acetate and glacial acetic acid (p.a.) were purchased from Sigma Aldrich (Vienna, Austria). For ultrapure water preparation, a Purelab Ultra system (ELGA LabWater, Celle, Germany) was used. Standards of *A. flavus* metabolites were collected from various research groups or purchased from the following commercial sources: Romer Labs^®^ Inc. (Tulln, Austria), Sigma–Aldrich (Vienna, Austria), Iris Biotech GmbH (Marktredwitz, Germany), Axxora Europe (Lausanne, Switzerland) and LGC Promochem GmbH (Wesel, Germany). Purchased standards were prepared for analysis as described by Sulyok et al. (2020) [33]. Trichloroacetic acid (TCA) and ascorbic acid (AA) were from Kemika (Zagreb, Croatia). Absolute ethanol was purchased from Panreac (Barcelona, Spain). Butylated hydroxytoluene (BHT) and 2-thiobarbituric acid (TBA) were obtained from Acros Organics (Morris Plains, NJ, USA). Stabilized 3% solution of hydrogen peroxide (H_2_O_2_) was obtained from Fluka (Steinheim, Germany), pyrogallol, and polyethylene glycol were from Merck (Darmstadt, Germany).

**Fullerol C_60_(OH)_24_ synthesis, preparation and characterization of nanoparticles solution**. The synthesis of fullerol C_60_(OH)_24_ [34], preparation of nanoparticle solution in ultrapure water and characterization of the particles by Dynamic Light Scattering (DLS) and Electrophoretic Light Scattering (ELS) techniques were performed as previously described by Kovač et al. (2017; 2018; 2020) [2,4,5]. The hydrodynamic size and the surface charge (zeta potential (ζ)) of the nanoparticle solution samples were determined by Zetasizer Nano ZS instrument (Malvern Instruments Inc., UK). All DLS analysis (633 nm wavelength and a measurement angle of 173^o^ (backscatter detection)) were performed in triplicates in aqueous solution at ambient temperature (25 °C) while zeta potential (ζ) measurements were performed in duplicates.

**Plant material, growth conditions, and drought-nanoparticle treatments**. For all experiments performed in this study, winter wheat genotype BC Tena was used in all experiments which was kindly provided by BC Institute, Rugvica, Croatia.

The effect of separate drought and fullerol C_60_(OH)_24_ nanoparticles (FNP) treatments as well as combined drought-nanoparticle treatments (FNP–PEG) was tracked for the wheat genotype. The drought was induced by 10% polyethylene glycol (PEG-6000) while the FNP concentrations were 10, 100, and 1000 ng mL^−1^ FNP. Combined treatments included 10 ng mL^−1^ FNP + PEG, 100 ng mL^−1^ FNP + PEG and 1000 ng mL^−1^ FNP + PEG, respectively. Seeds treated with distilled water represented control. Uniform seeds were sterilized in 70% ethanol for 10 min and washed three times with dH_2_O. One part of seeds were pre-soaked in FNP solutions and second part was immersed in distilled water for 24 h. Seeds were germinated in plastic pots (20 cm length × 15 cm width × 3 cm depth) on double-layered filter paper at 23 ± 2 °C/21 ± 2 °C (day/night). Pots were covered and regularly watered with 10 mL of distilled water or PEG solution. The seeds were kept in the dark for 2 days after which they were exposed to a light intensity of 80 μmol m^−2^ s^−1^ and 12 light/12 h dark photoperiod regime. Seeds whose coleoptile length reached 2 mm or more were considered to have germinated. Each set consisted of three pots per treatment with 100 seeds per pot.

**Determination of germination and morphometric parameters**. To evaluate the influence of separate drought and FNP treatments and combined drought–nanoparticle treatments on growth, germination percentage and shoot length were determined. The number of germinated seeds was recorded from 72 to 144 h while the shoot length was measured on 144 h. Germination percentage (%) for each day was calculated according to Gharoobi et al. (2012) [35]. Shoots were collected from control, drought, FNP treatments, and combined drought–nanoparticle treatments after 6 days and stored at −80 °C (Thermo Fischer Scientific Inc., TSE400VGP-ULTS, Waltham, MA, USA) prior to biochemical analyses.

Shoot extracts preparation for biochemical analyses. The biochemical analyses included the determination of activity of antioxidative enzymes and thiobarbituric acid reactive substances (TBARS) from lyophilized shoots. Previously frozen samples (−80 °C; Thermo Fisher Scientific Inc., TSE400VGP-ULTS, Waltham, MA, USA) were lyophilized (Christ, Alpha LSCplus, Osterode am Harz, Germany) under the following conditions; freezing temperature −55 °C; temperature of sublimation −35 °C to 0 °C; vacuum level 0.220 mbar. The temperature of isothermal desorption varied from 0 °C to 22 °C under the vacuum of 0.060 mbar. Freeze-drying lasted until the constant mass of mycelia was obtained, which was approximately 8 h.

For the enzyme activity measurements lyophilized tissue (20 mg) was homogenized in 1 mL ice cold 50 mM potassium phosphate extraction buffer (pH 7.0) containing 5 mM ascorbic acid, 0.1 mM ethylenediaminetetraacetic acid (EDTA) and 4% polyvinylpolypyrrolidone. Tissue was extracted using Omni^®^ Bead Ruptor 12 Homogenizer (Omni International, Kennesaw, GA, USA) at 6 m/s during 120 s with sample cooling on ice after every 20 s of disruption, by modified method as previously described by Kovač et al. (2019) [36]. After homogenization extracts were clarified by centrifugation (Thermo Scientific SL 8R; Thermo Scientific^TM^, Helsinki, Finland) at 15,000× *g* for 20 min at 4 °C. Obtained supernatants were used for the spectrophotometric measurements of antioxidative enzyme activities (Helios γ, Thermo Spectronic, Cambridge, UK).

Nonspecific peroxidase (POD; EC 1.11.1.7) was obtained by tracking the oxidation of guaiacol in the presence of H_2_O_2_ at 470 nm according to Siegel and Galston (1967) [37]. Reaction solution was the mixture of 0.2 mM potassium phosphate buffer (pH 5.8), 5 mM H_2_O_2_ and 5 mM guaiacol. Ascorbate peroxidase (APX; EC 1.11.1.11) activity was determined as decrease in absorbance of ascorbate at 290 nm [38]. Reaction occurred after adding 12 mM H_2_O_2_ to the reaction solution containing extraction buffer, 25 mM ascorbate acid and 0.1 mM EDTA. Catalase (CAT; EC 1.11.1.6) activity was analyzed according to Aebi (1984) [39] by the decline in absorbance as a result of H_2_O_2_ consumption at 240 nm. Reaction mixture consisted of extraction buffer and 5 mM H_2_O_2_. Polyphenol oxidase (PPO; EC 1.14.18.1) activity was recorded as increase of absorbance at 430 nm as a rate of oxidation of pyrogallol at 40 °C [40]. Reaction mixture (2 mL) contained 15 µL of extract, 100 mM potassium phosphate buffer (pH 7.0) and 100 mM pyrogallol. The activities of antioxidative enzymes was calculated as nkatals per gram fresh weight (nkatals gFW^−1^).

To determine the level of membrane damage under different drought, FNP and combined drought-nanoparticle treatment, thiobarbituric acid reactive substances (TBARS) in shoots was measured by a spectrophotometric assay at 535 nm [41]. Shoot extracts for TBARS assay were prepared as described above, but with additional 100 mg/mL of TCA. Prepared extract (500 μL) was mixed with 1 mL of TBA reagent (saturated TBA (γ = 0.67 mg mL^−1^), 0.1 M HCl and 10 mM BHT, pH 2.5). Control samples contained 500 μL of ultrapure water instead of extracts. After heating at 95 °C for 60 min, samples were rapidly cooled in an ice bath and followed by an addition of equal volume of n-butanol, vigorous mixing, and incubation for 10 min at 25 °C. Butanol phase containing TBARS was separated from water phase by centrifugation at 2795× *g* for 10 min at 25 °C using a Centric 150 centrifuge (Tehtnica, Železniki, Slovenia), and used for spectrophotometric evaluation of TBARS concentration. Molar extinction coefficient of malonyldialdehyde (ε535 nm = 156 × 103 M^−1^ cm^−1^) was used for the calculation of TBARS concentration.

**Cultivation of *Aspergillus flavus* NRRL 3251 on mycological media**. *Aspergillus flavus* NRRL 3251 culture maintained on malt extract agar at 4 °C was used in this study. The *A. flavus* was grown on potato dextrose agar in the dark at 29 °C for 7 days to stimulate conidia production, as previously described [2].

Wheat samples pre-treatment with FNP and inoculation with *A. flavus* conidia-unappropriated storage conditions simulation and impact on secondary metabolite profile. The impact of FNP on *A. flavus* secondary metabolism was tracked for 168 h, during a simulation of inappropriate storage conditions. The inoculation of wheat samples was conducted in triplicates in Petri dishes. The 10 g of FNP pre-treated seeds, as previously described, was placed on two layers of filter paper sodden with 1 mL of distilled water and inoculated by 200 µL of conidia suspension (2.5 × 10^6^) and incubated in Thermostatic cabinet (Aqualytic^®^, AL 500-8, Langen, Germany) in the dark at 29 °C.

The metabolites produced by *A. flavus* during growth on FNP-pre-treated wheat samples were determined by the multi-analyte “dilute and shoot” LC-MS/MS method of Sulyok et al. (2020) [33]. For the analysis, 5 g of the infected wheat seeds were mixed with 20 mL of extraction solvent (acetonitrile/water/acetic acid 79:20:1, *v/v/v*) and extracted for 90 min at ambient temperature using GLF 3017 rotary shaker (GLF, Germany). After extraction, 500 µL of the extracts were transferred into glass vials and diluted with 500 µL of dilution solvent (acetonitrile/water/acetic acid 20:79:1, *v/v/v*). Vial contents were vigorously mixed and 5 µL was injected directly into the LC-MS/MS system. The screening and detection of metabolites was performed as described by Malachová et al. (2014) [42], and in brief the QTrap 5500 MS/MS detector (Applied Biosystems, Foster City, CA, USA) equipped with TurboV electrospray ionization (ESI) source, and Agilent 1290 binary UHPLC system (Agilent Technologies, Waldbronn, Germany) were used. For the separation of the metabolites, C18 security guard pre-column (4 × 3 mm i.d.) (Phenomenex, Torrance, CA, US) with the Gemini^®^ C18 column (150 × 4.6 mm i.d., 5 µm particle size) was used. Freshly prepared eluents and the gradient were exactly as described by Malachová et al. (2014) [42]. The Scheduled selected reaction monitoring (sSRM) mode was applied, and two runs per sample were used (each for one mode). The detection window was set to ±27 s in positive and ±42 s in negative mode due to high number of monitored metabolites. The ESI source parameters were exactly as described by Malachová et al. (2014) [42]. At least two sSRM transitions were monitored per metabolite (quantifier and qualifier), and according to the validation guidelines the ratio between two transitions were used as additional identity confirmation point.

***Aspergillus flavus* aggressiveness on wheat infection under FNP impact**. The Petri-dish test according to Purahong et al. [43] using Area Under Healthy tissue Progress Curve (AUHPC) and Standardized Area Under Disease Progress Curve (AUDPC_standard_) was used to determine the aggressiveness of *A. flavus* NRRL 3251 in presence of FNP. Moreover, 25 seeds were sterilized and pre-treated by FNP solutions as previously described and inoculated with *A. flavus* conidia suspension (1 × 10^6^; 10 mL), placed in pots with the embryo turned upwards on sterile double-layer filter paper. Pots were incubated at 29 °C and germinated seeds were counted 48 h after inoculation and this value was taken as 100% germination and the healthy coleoptiles were counted every day from 72 to 144 h of incubation period. The AUHPC was calculated as sum of the percentage of healthy coleoptile from 48 to 144 h (AUHPC = (B_1_ + B_2-5_)/2); values from 50 (very aggressive); all seedlings diseased at 72 h; up to 400 (not aggressive); no diseased seedlings. When AUHPC was transformed to AUDPC_standard_, ((400-AUHPC)/350); the values ranged from 0 (not aggressive) to 1 (very aggressive).

**Statistical analysis**. The results were obtained from three independent FNP treatments and three FNP treatments combined with drought in three pots per treatment. For the determination of biochemical changes, samples were collected from each pot, pooled, and divided in three technical repetitions per treatment. For statistical analysis three repetitions were used. In all experiments factorial analysis of variance (ANOVA) was performed in order to reveal the effect of treatment (T), FNP treatments (N), and combined T × N Tukey’s post-hoc test was conducted for presentation of the significant differences among the mean values. Principal Component Analysis (PCA) was used to discriminate morphological changes to different treatment combinations. Eight variables were included in PCA analysis. Factor loadings were performed to estimate the proportion of total variance with different principal components (PC). The loadings showed correlations between different principal components and variables where high loadings with values >0.71 were considered as strong correlation [44]. Spearman’s correlation coefficients, R were calculated for metabolic profile of *A. flavus* and aggressiveness of *A. flavus* in infected pretreated FNP wheat, uninfected FNP wheat and controls. Statistical analyses were performed using the Statistica 13.5 (TIBCO Software Inc., Palo Alto, CA, USA).

## 3. Results

### 3.1. Fullerol C_60_(OH)_24_ Nanoparticles Characterization

**Figure 1:**
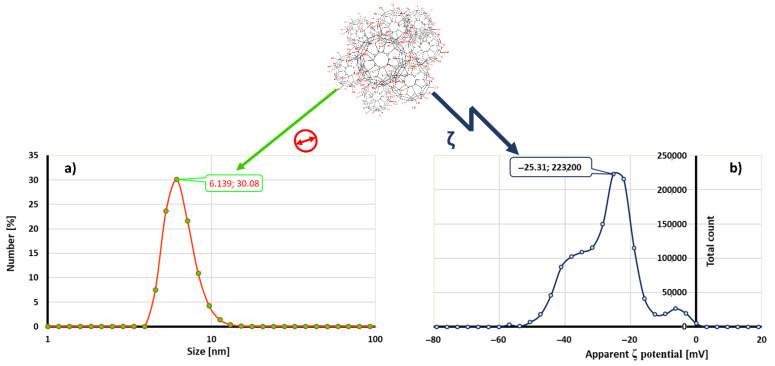
Fullerol C_60_(OH)_24_ aqueous nanoparticles solution (c = 10 mg mL^−1^) size distribution by number (**a**) and apparent zeta potential (ζ) (**b**). Data represent one selected result out of three measurements with similar obtained values of mean hydrodynamic radius (**a**) and surface charge (**b**). 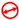
- diameter of the fullerol C_60_(OH)_24_ aqueous nanoparticles.

The results of FNP characterization was obtained by Dynamic Light Scattering (DLS) and Zeta (ζ) potential measurements (Figure 1). FNP aqueous solution had particles with sizes less than 10 nm as most dominant, with a tendency to form bigger agglomerates with dimensions under 100 nm. Figure 1a represents the results of particle size distribution by number, showing that most of the FNP had the mean hydrodynamic radius in the range of 4–11 nm, with most of the particles (30.08%) with a size of 6 nm. Moreover, ζ potential measurements confirmed that the mean value of analyzed FNP was −25.31 mV (Figure 1b).

### 3.2. Fullerol C_60_(OH)_24_ Nanoparticles and Drought Simulation Impact on Morphometric Parameters of the BC Tena Wheat Genotype

**Figure 2:**
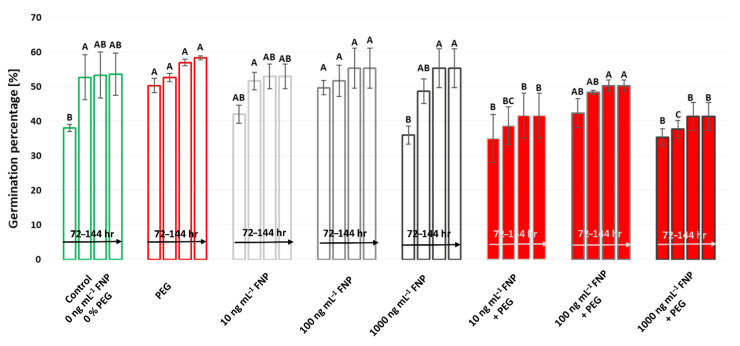
Germination percentage under drought (PEG), fullerol C_60_(OH)_24_ nanoparticles (FNP; 10, 100 and 1000 ng mL^−1^) and combined FNP–PEG treatments (10 ng mL^−1^ FNP + PEG, 100 ng mL^−1^ FNP + PEG, and 1000 ng mL^−1^ FNP + PEG) during 72, 96, 120, and 144 h (from **left**–**right**). Germination percentage is calculated for period of 72 to 144 h. Values are means ± S.D. (*n* = 3). The different letters indicate statistically significant differences among the mean values of different treatments at *p* < 0.05 using Tukey’s post-hoc test.

The impact of FNP and drought on the germination of wheat genotype is presented in Figure 2. There were no significant changes in the germination between control and FNP treatments except at 100 ng mL^−1^ of FNP, when germination was promoted after 72 h of the examined period but this effect was lost over time. Surprisingly, PEG and 100 ng mL^−1^ FNP treatments had a more positive effect on the germination than control or the highest FNP concentration (1000 ng mL^−1^) at the earliest time point. The combination of FNP treatments with drought (10 and 1000 ng mL^−1^ FNP + PEG) showed inhibitory effect on the germination, in comparison to single PEG and FNP treatments at the same concentration (10 and 1000 ng mL^−1^).

**Figure 3:**
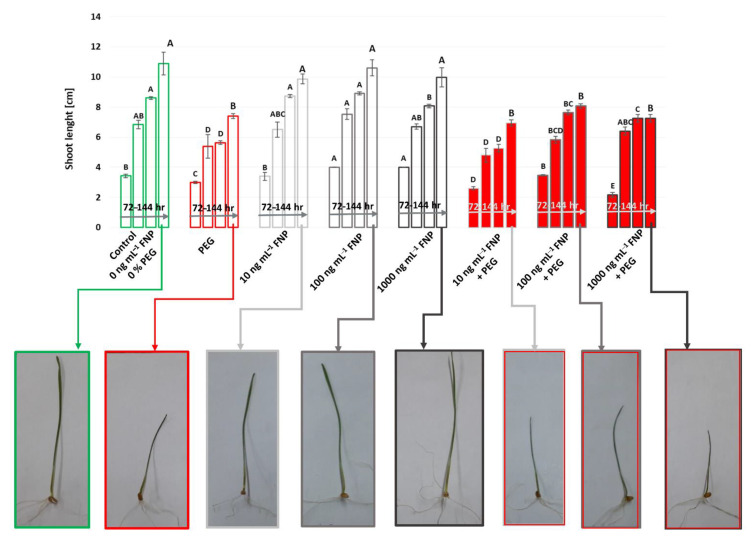
Shoot length under drought (PEG), fullerol C_60_(OH)_24_ nanoparticles (FNP; 0, 10, 100 and 1000 ng mL^−1^) and combined FNP–PEG treatments (10 ng mL^−1^ FNP+ PEG, 100 ng mL^−1^ FNP + PEG, and 1000 ng mL^−1^ FNP + PEG) during 72, 96, 120 and 144 h (from left–right). Shoot length is measured from period of 72 to 144 hr. Photos represent average shoot length for all treatment after 144 hr. Values are means ± S.D. (*n* = 3). The different letters indicate statistically significant differences among the mean values of different treatments at *p* < 0.05 using Tukey’s post-hoc test.

The effect of FNP and drought on the shoot growth of wheat genotype is presented in Figure 3. Drought significantly impaired the shoot growth in respect to optimal conditions. The effect of FNP treatments on shoot length was recorded only after 72 h. FNP at higher concentrations (100 and 1000 ng mL^−1^) increased shoot length compared to control (0 ng mL^−1^ of FNP) or 10 ng mL^−1^ of FNP, respectively. Benefits caused by FNP treatments were more evident under drought. All FNP treatments improved the shoot growth from 96 to 144 h in comparison to PEG treatment. Moreover, the growth of shoots exposed to single FNP treatments increased over time compared to all FNP treatments combined with drought. These results showed that nanoparticles in combination with drought were more hazardous for the plant.

**Figure 4:**
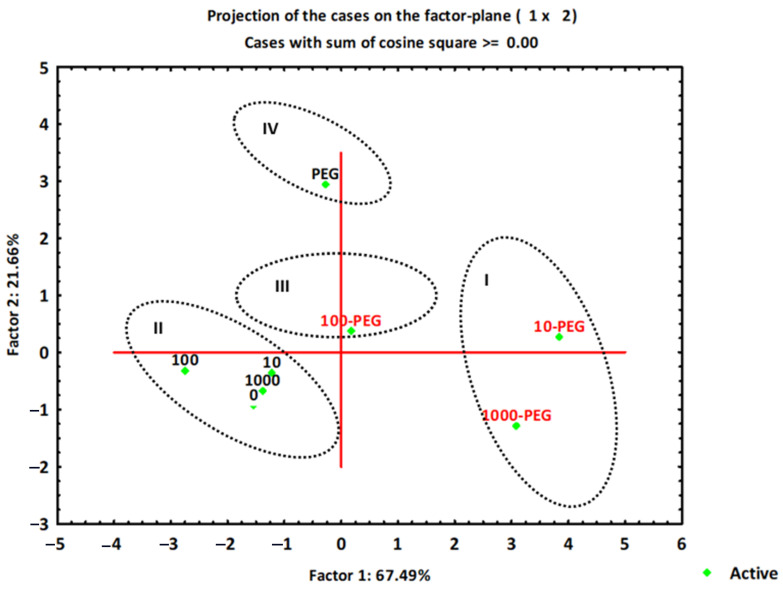
Principal component analysis (PCA) of data sets of morphometric parameters under drought (PEG), fullerol C_60_(OH)_24_ nanoparticles (FNP; 10, 100, and 1000 ng mL^−1^) and combined FNP–PEG treatments (10 ng mL^−1^ FNP + PEG, 100 ng mL^−1^ FNP + PEG, and 1000 ng mL^−1^ FNP + PEG). Scores of the first two factors. Black letters present control; red letters present drought. PCA was used to distinguish the effect of single treatment application (control, drought or nanoparticles) and combined drought-nanoparticle treatment. Values are means ± S.D. (*n* = 3). The different letters indicate statistically significant differences among the mean values of different treatments at *p* < 0.05 using Tukey’s post-hoc test. **▪** Active data.

PCA analysis showed changes in morphometry parameters that occurred under exposure of wheat to single treatments (PEG or FNP) and under combined FNP–PEG treatments (10 ng mL^−1^ FNP + PEG, 100 ng mL^−1^ FNP+ PEG, and 1000 ng mL^−1^ FNP+ PEG) during the examined period. The PCA yielded the two most important principal components (PC1 and PC2) describing 89.2% of data variation (Figure S1). The total variation of first principal component (PC1) was 67.5% and second principal component was 21.7% of data variation (Table S1). PC1 was determined by negative loadings on most of the morphometric variables except for germination at 72 h of the examined period. Score plots revealed four clusters (Figure 4). Cluster I included two treatments (10 ng mL^−1^ FNP + PEG and 1000 ng mL^−1^ FNP + PEG) due to a decrease in germination and shoot length. Control and FNP treatment groups (10, 100, and 1000 ng mL^−1^) showed both a high germination percentage and increased shoot length; for that reason they are separated as cluster II. Combined 100 ng mL^−1^ FNP + PEG treatment segregated as cluster III due to increased germination at the 72 h point and exhibited decreased shoot growth compared to control and PEG, while cluster IV discriminated PEG treatment as a separate group due to reduced shoot growth and germination. Factor loadings are presented in Table S1.

Analysis of variance of morphologic parameters revealed significant treatment (T), nanoparticles (N) and T × N effect for most parameters. Exceptions include germination after 72 h (G3), which was independent of the treatment (T), and shoot length (S6), which did not show T × N interactions at the last day of the experiment (Table S2).

### 3.3. Antioxidative Enzymes Activity and TBARS Content in Shoot Extracts

The impact of single FNP and drought treatment and combined FNP-drought treatments on antioxidative enzymes activity and TBARS content on the shoots is presented in Table 1. Among all antioxidative enzymes, only POD showed higher activity under drought compared to control. Furthermore, POD activity was also increased under higher FNP concentration (100 and 1000 ng mL^−1^) in regards to control and lower FNP concentration. In the presence of PEG the highest and the lowest FNP concentrations (10 ng mL^−1^ FNP + PEG and 1000 ng mL^−1^ FNP + PEG) induced POD activity, compared to their respective groups (PEG, 10 and 1000 ng mL^−1^ of FNP). Conversely, 100 ng mL^−1^ FNP + PEG treatment showed a decline in POD activity in regards to single PEG, 100 ng mL^−1^ FNP and other combined FNP–PEG treatments. Under 1000 ng mL^−1^ FNP + PEG, APX activity remarkably increased compared to other corresponding group treatments. Combined FNP-drought treatments had an inhibitory effect on CAT activity in comparison to control and respective FNP treatments while PPO activity did not show significant changes in all treatments. Analysis of variance of biochemical parameters presented strong interactions between treatments (T), nanoparticles (N) and T × N for most traits. The interactions were not found in case of PPO activity which was independent of the treatment (T) and T × N and for TBARS whose content was T × N independent (Table S3).

The independent application of PEG or FNP treatments had a negative effect on the membrane integrity. TBARS content was significantly increased under PEG treatment and the highest FNP concentrations (100 and 1000 ng mL^−1^) compared to control (Table 1). Significant changes between PEG and combined FNP–PEG treatments were not detected. However, the combined treatments resulted in higher membrane damage. Combined treatments (10 ng mL^−1^ FNP+ PEG and 1000 ng mL^−1^ FNP+ PEG) had higher TBARS content compared to the corresponding FNP group.

### 3.4. Aspergillus Flavus Aggressiveness Test in the Presence of Fullerol C_60_(OH)_24_ Nanoparticles

The aggressiveness of *A. flavus* on FNP treated wheat seeds is presented in Table 2. The results depicted that FNP pre-treatment of wheat caused a decrease in *A. flavus* aggressiveness in a concentration dependent pattern. The lowest applied FNP concentration provoked the strongest response of *A. flavus* aggressiveness; however not at a statistically significant level.

### 3.5. Aspergillus Flavus NRRL 3251 Secondary Metabolite Profile after the Infection of Wheat Seeds Pre-Treated with Fullerol C_60_(OH)_24_ Nanoparticles

The *A. flavus* secondary metabolite profile after the infection of FNP pre-treated BC Tena wheat genotype is presented in Table 3. The analysis revealed six metabolites that belonged to 3-nitropropionic acid, aflatoxins B1 and B2 (AFT B1; AFT B2), aflatoxicol, O-methylsterigmatocystin, and kojic acid (Table 3). According to the results, the tested wheat genotype was not contaminated with mycotoxins, except in the case of 3-nitropropionic acid which was found in all treatment combinations. Comparing treatments under *A. flavus* infection, concentration of 3-nitropropionic acid was 2-fold higher at 100 ng mL^−1^ FNP than at control treatment (0 ng mL^−1^ FNP), while at 1000 ng mL^−1^ FNP its amount reached values up to 69 times higher. Moreover, at the highest level of FNP, the concentration of 3-nitropropionic acid was 21 times higher compared to 100 ng mL^−1^ FNP under *A. flavus* infection. In addition, when comparing the control and inoculated sample there were 1.8 times higher levels of 3-NPA in inoculated samples. On the other hand, the concentrations of 3-NPA are 0.85 times lower in the non-infected sample with 1000 ng mL^−1^ of FNP indicating that FNP can reduce 3-NPA if there is no live fungi on wheat. For the AFT B1 production significant differences existed between infected control (0 ng mL^−1^ FNP), 10 and 100 ng mL^−1^ FNP. Control sample exhibited 1 to 26 % higher production, compared to 100 ng mL^−1^ FNP and 10 ng mL^−1^ FNP, respectively. Treatment of 100 ng mL^−1^ FNP under *A. flavus* infection showed the largest value of kojic acid than all applied treatments (7880% higher kojic acid production).

## 4. Discussion

Before setting new goals in the attempt to establish the mechanism of action of FNP during interaction with mycotoxigenic fungi, it is essential to conduct experiments to observe the influence of FNP on wheat seeds, as one of the important substrates for the growth of such microorganisms. Moreover, the influence of drought should be considered in such an experiment, as it is a major factor of global climate change, which has a high impact on the environment. Accordingly, the aim of this study was to determine the effect of FNP on wheat seed germination and shoot length, both in and without combination with drought, the effect on plant cell oxidative status and its impact on *A. flavus* aggressiveness during wheat germination, and the effect on secondary metabolite profile during wheat storage under unsuitable storage conditions. Such reports are not currently available in the scientific literature, but it will be an important step for future studies and will support the investigation of the mechanism of action of FNP during interaction with mycotoxigenic fungi.

The theoretically calculated FNP molecule radius is 1 nm, and the data in this study again confirmed the high tendency of FNP to form agglomerates, whereas there were non-agglomerated (single) FNP molecules detected by DLS measurements (Figure 1a). Measurements were conducted for the purpose of the surface charge assessment (Figure 1b). The obtained results indicate that prepared FNP water suspension is a stable polyanionic system (ζ = −25.31), which is composed of clusters with sizes less than 100 nm (6 nm). Such results were in accordance with EC recommendation for the definition of the term “nanomaterial” and our previously published results [2,4,5,27,45,46,47].

Carbon nanoparticles can penetrate across the seed coat and accelerate the rate of water imbibition, which has an influence on germination and growth [48]. Morphometry analysis revealed the positive effect of higher nanoparticle treatments (100 or 1000 ng mL^−1^ FNP) in the earliest time point on the germination and shoot growth compared to control conditions (Figure 2 and Figure 3). PCA analysis also shows the same (Figure 4). Stimulated germination and growth as a result of functionalized carbon nanotubes (CNTs) treatment was also recorded in tomato seeds [49] and barely root [29]. Our results do not support the study of Wang et al. (2016) [13], which showed that fullerol particles in wheat are mostly retained in the root system with a low rate of translocation to shoots. In the same study, fullerol promoted root development and chlorophyll synthesis but did not promote shoots. Surprisingly, a different scenario occurred under drought. The germination was higher under PEG treatment than at 1000 ng mL^−1^ FNP treatment which implies a suppressing role of higher nanoparticle concentration (Figure 2). Another reason could be related to the stimulation of different water channels (aquaporins, AQPs) on the seed coat. According to Burman and Kurman (2018) [50], carbon nanoparticles (CTNs) and osmotic stressors use different mechanisms for the gating of AQPs. In tomato, CTNs caused higher induction of *LeAqp2* gene [49]. In the same study, a multiwall-CNT treatment caused the stimulation of AQPs, thereby activating the signaling pathways which modify the cell wall and promote growth. However, the expression of AQPs under drought is not so simple. There are still lots of disagreements whether their activity is upregulated or downregulated [51]. On the other hand, shoots were longer under FNP treatments than under drought which leads on conclusion that application of nanoparticles depends not only on concentration but also on the growth stage. The connection between engineered nanomaterials and plant growth stage was also presented in some studies [52].

Drought alters the physiological and biochemical processes and contributes to ROS overproduction which endangers the membrane integrity and activity of antioxidative enzymes [53]. Compared to control, the inhibitory effect of drought was manifested in decreased shoot length and increased TBARS content while germination percentage and antioxidative enzymes remained unchanged (Table 1, Figure 1). In this study, the mild drought level for the wheat was used. Tena is a drought tolerant genotype (unpublished results) so such low drought intensity perhaps had a stimulating effect on germination. According to literature, PEG application in some cases can improve water imbibition and speed up the germination [54,55].

In plant physiology, the interaction of FNP with different abiotic stresses is still a poorly explored area. However, the dual nature of fullerene and its derivatives in reactive oxygen species (ROS) removal or their synthesis is well documented [56]. In our study, the effect of FNP under PEG-induced drought was observed. A combined FNP + PEG treatment inhibited the germination (1000 ng mL^−1^ FNP + PEG) and the shoot growth in contrast to single FNP application. Moreover, TBARS level increased and activity of CAT, POD and APX decreased under combined FNP + PEG treatments. The higher membrane damage could be due to ROS overproduction, which inhibits the enzyme’s antioxidative defense mechanisms. The same negative effect of fullerol was also noticed in similar studies. In tobacco BY−2 cells, the overproduction of ROS and higher membrane damage was evident after exposure to carboxyfullerene treatment [57]. On the other hand, fullerol provided drought tolerance in maize and *Brassica napus* by reducing the ROS level and malondialdehyde (MDA) [28,58]. Regarding this, the protective role of fullerol nanoparticles in drought-related studies depends on plant species and concentration and should be interpreted with caution. Considering this, it is evident that simultaneous FNP and drought appearance will cause more damage for the plant than stress provoked by FNP or drought alone. The principle of action of FNP and PEG on the plant water regime is opposite. While FNP treatment promotes the water status of the plant, at the same time PEG treatment limits the water intake. Based on this we could infer that drought in combination with negatively charged FNP amplifies a phytotoxicity of FNP since they can easily cross the cell barrier [50,52].

*A. flavus* aggressiveness test in the presence of FNP was carried out to quantify the FNP influence upon infection of natural substrates like wheat seeds (Table 2). The FNP pre-treatment of the wheat caused a decrease of *A. flavus* aggressiveness (AUDPC_standard_) in the concentration dependent pattern; the lowest concentration provoked the strongest aggressiveness. Despite the absence of statistically significant difference, the intriguing question—occurrence and impact of future possible FNP higher concentrations—remains for further studies. Unfortunately, there is no literature data that can be compared to our results and a study which includes more mycotoxigenic fungi species should be conducted to establish a solid opinion on FNP impact on foodborne mycotoxigenic fungi aggressiveness. The observed AUDPC_standard_ values were not in correlation with measured concentration of *A. flavus* secondary metabolites (Table S4). This depicts the different effect of FNP on wheat seeds during germination process in comparison with the effect observed during *A. flavus* infection of wheat seeds that are not stored properly.

In general, the secondary metabolites biosynthesis was influenced by FNP (Table 3). At a certain concentration all detected metabolites were produced after infection of control samples of wheat seed, except aflatoxicol which was produced when FNP were applied. The 3-nitropropionic acid concentration was increased when FNP applied concentration was increased. The same effect was observed for AFT B1, while the opposite occurred with aflatoxicol which is an AFT B1 precursor. Furthermore, pre-treatment of wheat with 100 ng mL^−1^ of FNP provoked the highest O-methylsterigmatocystin and kojic acid concentration. The mentioned differences are statistically significant (Table 3), except in the case of aflatoxicol. Moreover, correlations between produced secondary metabolites were observed (Table S4). The 3-nitropropionic acid concentrations were in correlation with produced concentrations of AFT B1 (*p* = 0.01), kojic acid (*p* = 0.01), and O-methylsterigmatocystin (*p* = 0.05). AFT B1 concentration was in correlation with concentration of AFT B2 (*p* = 0.05), while AFT B2 correlated with O-methylsterigmatocystin concentrations (*p* = 0.01).

## 5. Conclusions

In conclusion, higher FNP treatment (100 and 1000 ng mL^−1^) showed a beneficial effect on germination and shoot length compared to the control. Opposite to that, germination was more reduced under 1000 ng ml^−1^ FNP than under drought which can tentatively be explained by the harmful effect of higher nanoparticle concentrations at early growth stage. Under combined treatments (FNP + PEG) the germination, shoot growth and activity of CAT, POD, and APX decreased and TBARS content increased compared to single FNP application, indicating that co-occurrence of drought and FNP is more detrimental for the existence of the plant in nature. Moreover, FNP pre-treatment of wheat caused a concentration dependent decrease in the aggressiveness of *A. flavus*. The data suggest that FNP may cause the increased production of *A. flavus* secondary metabolites during wheat storage under inappropriate conditions. Underlying this is increased production of 3-nitropropionic acid and kojic acid, but also production of aflatoxicol in FNP-pre-treated samples. One of the possible reasons for such FNP effect could be its ability to modulate oxidative status, both in fungal and plant cells. The presented results indicate that the interaction among FNP–wheat–*A. flavus* becomes even more complex under drought conditions.

Furthermore, this study should be the driving factor for further follow-up studies on the same topic, with the results determining the direction and hypotheses for further research. It will be an important step to reveal the exact mechanism of FNP effect and its influence on food and feed safety.

## Figures and Tables

**Table 1:** Activity of antioxidative enzymes and TBARS content under drought (PEG), fullerol C_60_(OH)_24_ nanoparticles (FNP; 0, 10, 100, and 1000 ng mL^−1^) and combined FNP–PEG treatments (10 ng mL^−1^ FNP + PEG, 100 ng mL^−1^ FNP + PEG, and 1000 ng mL^−1^ FNP + PEG).

*Treatment*	*APX*	*CAT*	*POD*	*PPO*	*TBARS [pmol/g]*
	*(nkat/g DW)*				
*Control; 0 ng mL^−1^ FNP*	*0.1 ± 0.1 bc*	*152.9 ± 12.2 ab*	*3.3 ± 0.4 d*	*1.0 ± 0.0 a*	*6.2 ± 2.1 c*
*PEG*	*0.8 ± 0.1 bcd*	*168.3 ± 27.5 a*	*4.5 ± 0.9 c*	*1.1 ± 0.0 a*	*57.7 ± 6.6 a*
*10 ng mL^−1^ FNP*	*0.6 ± 0.08 de*	*165.1 ± 24.4 a*	*2.9 ± 0.0 d*	*1.1 ± 0.0 a*	*18.6 ± 3.5 c*
*100 ng mL^−1^ FNP*	*0.6 ± 0.1 cde*	*110.1 ± 0.0 bc*	*4.4 ± 0.7 c*	*0.9 ± 0.1 a*	*51.3 ± 1.0 ab*
*1000 ng mL^−1^ FNP*	*1.1 ± 0.0 b*	*99.7 ± 13.7 cd*	*6.1 ± 0.1 b*	*1.2 ± 0.3 a*	*44.6 ± 6.2 b*
*10 ng mL^−1^ FNP+ PEG*	*0.3 ± 0.0 e*	*89.7 ± 9.3 cd*	*6.2 ± 0.6 b*	*1.1 ± 0.0 a*	*56.8 ± 0.2 a*
*100 ng mL^−1^ FNP+ PEG*	*0.7 ± 0.0 bcd*	*63.0 ± 9.4 de*	*3.1 ± 0.3 d*	*1.0 ± 0.1 a*	*67.1 ± 1.0 a*
*1000 ng mL^−1^ FNP+ PEG*	*1.9 ± 0.3 a*	*30.6 ± 6.1 e*	*7.4 ± 0.2 a*	*1.1 ± 0.0 a*	*74.9 ± 12.2 a*

Values are means ± S.D. (*n* = 3). The different letters indicate statistically significant differences between treatments at *p* < 0.05, using Tukey’s post-hoc test. APX—ascorbate peroxidase (EC 1.11.1.11); CAT—catalase (EC 1.11.1.6); POD—nonspecific peroxidase (EC 1.11.1.7); PPO—polyphenol oxidase (EC 1.14.18.1); TBARS—thiobarbituric acid reactive substances.

**Table 2:** Means of area under healthy tissue progress curve (AUHPC) and standardized area under disease progress curve (AUDPC standard) from Petri-dish test after inoculation of fullerol C_60_(OH)_24_ nanoparticles (FNP) pre-treated wheat genotype BC Tena with *Aspergillus flavus* NRRL 3251.

Treatment	AUHPC	AUDPC_Standard_
*Control, 0 ng mL^−1^ FNP + A. flavus*	*243 ± 13.7 a*	*0.45 ± 0.04 a*
*10 ng mL^−1^ FNP + A. flavus*	*212 ± 24.4 a*	*0.54 ± 0.07 a*
*100 ng mL^−1^ FNP + A. flavus*	*260 ± 13.2 a*	*0.40 ± 0.04 a*
*1000 ng mL^−1^ FNP + A. flavus*	*275 ± 11.7 a*	*0.36 ± 0.03 a*

Values are represented as means ± SEM (*n* = 3). The different letters indicate statistically significant differences between treatments at *p* < 0.05, using Tukey’s post-hoc test.

**Table 3:** The *A. flavus* secondary metabolite profile after the infection of fullerol C_60_(OH)_24_ nanoparticles (FNP) pre-treated (0, 1, 10, 100, and 1000 ng mL^−1^) BC Tena wheat genotype. Data expressed as ng mL^−1^ represent the mean ± SEM from three separate experiments.

Treatment	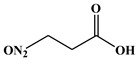 3-Nitropropionic acid	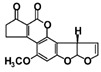 Aflatoxin B1	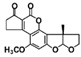 Aflatoxin B2	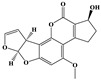 Aflatoxicol	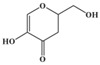 Kojic acid	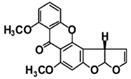 O-Methylsterigmatocystin
	(ng mL^−1^)					
Control; 0 ng mL^−1^ FNP	*9.01 ± 0.13 cd*	*<LOD d*	*<LOD b*	*<LOD a*	*<LOD b*	*<LOD b*
Control, 0 ng mL^−1^ FNP + *A. flavus*	*25.5 ± 0.09 c*	*6.63 ± 0.06 A*	*0.07 ± 0 a*	*<LOD a*	*16.8 ± 0.32 b*	*0.17 ± 0 a*
10 ng mL^−1^ FNP	*7.95 ± 0.30 cd*	*<LOD D*	*<LOD b*	*<LOD a*	*<LOD b*	*<LOD b*
100 ng mL^−1^ FNP	*13.9 ± 0.29 cd*	*<LOD D*	*<LOD b*	*<LOD a*	*<LOD b*	*<LOD b*
1000 ng mL^−1^ FNP	*1.33 ± 0.06 d*	*<LOD D*	*<LOD b*	*<LOD a*	*<LOD b*	*<LOD b*
FNP 10 ng mL^−1^ FNP + *A. flavus*	*5.03 ± 0.22 cd*	*4.94 ± 0.13 C*	*0.01 ± 0 b*	*0.37 ± 0.16 a*	*30.6 ± 0.20 b*	*0.11 ± 0.04 ab*
FNP 100 ng mL^−1^ FNP + *A. flavus*	*79.6 ± 0.07 b*	*5.83 ± 0.12 B*	*0 ± 0 b*	*0.35 ± 0.04 a*	*1338 ± 189 a*	*0.15 ± 0 a*
FNP 1000 ng mL^−1^ FNP + *A. flavus*	*1776 ± 10.8 a*	*6.77 ± 0.43 A*	*0.02 ± 0.02 b*	*0.19 ± 0.15 a*	*51.7 ± 2.23 b*	*0.11 ± 0.04 ab*

Values are represented as means ± SEM (*n* = 3). The different letters in each column indicate statistically significant differences between treatments at *p* < 0.05, using Tukey’s post-hoc test. <LOD—below detection limit. The text following an equation need not be a new paragraph. Please punctuate equations.

## Data Availability

The data presented in this study are openly available.

## Supplementary Materials

The following are available online at https://www.mdpi.com/2309-608X/7/3/236/s1, Table S1: factor loadings of morphometric parameters. Principle component analysis (PCA) was applied for evaluation of morphometric parameters under control (0), drought (PEG), nanoparticle (10, 100 and 1000 ng mL^−1^ FNP) and combined drought-nanoparticle treatments (10-PEG, 100-PEG and 1000-PEG). Data were analyzed by using STATISTICA 13.5 software package. Table S2: analyze of variance of morphometric parameters under control (0), drought (PEG), nanoparticle (10, 100, and 1000 ng mL−1 FNP) and combined drought-nanoparticle treatments (10-PEG, 100-PEG and 1000-PEG). Data were analyzed by using factorial ANOVA in STATISTICA 13.5 software package. Table S3: analyze of variance of biochemical parameters under control (0), drought (PEG), nanoparticle (10, 100 and 1000 ng mL^−1^ FNP) and combined drought-nanoparticle treatments (10-PEG, 100-PEG and 1000-PEG). Data were analyzed by using factorial ANOVA in STATISTICA 13.5 software package. Table S4: correlation among metabolic profile of *A. flavus* and *A. flavus* aggressiveness under different pretreatments and treatments. Spearman’s correlation coefficients (R) were calculated for metabolic profile of *A. flavus* and aggressiveness of *A. flavus* in infected pretreated FNP seeds, uninfected FNP seeds and control using STATISTICA 13.5. Figure S1: principal component analysis of data sets of morphometric parameters under control (0), drought (PEG), nanoparticle (10, 100 and 1000 ng mL^−1^ FNP) and combined drought-nanoparticle treatments (10-PEG, 100-PEG and 1000-PEG). Loadings of first two factors. S3-S6 (shoot length from 3–6 days) and G3-G6 (germination from 3–6 days). PCA was used to distinguish the effect of single treatment application (control, drought or nanoparticles) and combined drought-nanoparticle treatment. Data were analyzed by using STATISTICA 13.5 software package.
