# Supplementary material for: Fullerol C60(OH)24 Nanoparticles and Drought Impact on Wheat (Triticum aestivum L.) during Growth and Infection with Aspergillus flavus
Source: J Fungi (Basel). 2021 Mar 22;7(3):236. doi: 10.3390/jof7030236 (PMC8004204; doi:10.3390/jof7030236)
Supplement: Supplementary file 1 [file jof-07-00236-s001.pdf]

Table S1. Factor loadings of morphometric parameters. Principle component analysis (PCA) was applied for evaluation of morphometric parameters under control (0), drought (PEG), nanoparticle (10, 100 and 1000 ng mL^{-1} FNP) and combined drought-nanoparticle treatments (10-PEG, 100-PEG and 1000-PEG). Data were analysed by using STATISTICA 13.5 software package.

Variable	PC1	PC2
G3	-0.56	0.65
G4	-0.92	0.31
G5	-0.92	0.36
G6	-0.89	0.41
S3	-0.89	-0.08
S4	-0.73	-0.58
S5	-0.73	-0.63
S6	-0.86	-0.44
Explained variance (eigenvalue)	5.40	1.73
Proportion of total variance (%)	67.49	21.66
Cumulative variance (%)	67.49	89.15

PC-1 (principal component 1); PC-2 (principal component 2); G3-G6 (germination from 3-6 days), S3-S6 (shoot lenght from 3-6 days).

Table S2. Analyse of variance of morphometric parameters under control (0), drought (PEG), nanoparticle (10, 100 and 1000 ng mL^{-1} FNP) and combined drought-nanoparticle treatments (10-PEG, 100-PEG and 1000-PEG). Data were analysed by using factorial ANOVA in STATISTICA 13.5 software package.

Source of variation	Df	MS							
		G3	G4	G5	G6	S3	S4	S5	S6
Treatment (T)	1	2.70	280.2*	266.7*	247.1*	4.9*	9.9*	27.1*	51.0*
Nanoparticle (N)	3	139.4*	113.8*	82.5*	97.7*	0.6*	1.3*	2.1*	1.2*
T x N	3	126.8*	57.2*	92.6*	103.3*	0.6*	0.7*	2.5*	0.3 ns
Error		11.9	15.0	23.1	22.2	0.02	0.2	0.03	0.2

* significant at P≤0.05; ns-not significant.

Table S3. Analyse of variance of biochemical parameters under control (0), drought (PEG), nanoparticle (10, 100 and 1000 ng mL^{-1} FNP) and combined drought-nanoparticle treatments (10-PEG, 100-PEG and 1000-PEG). Data were analysed by using factorial ANOVA in STATISTICA 13.5 software package.

Source of variation	Df	MS				
		APX	CAT	POD	PPO	MDA
Treatment (T)	1	0.1*	11663.7*	7.3*	0.01 ns	4612.4*
Nanoparticle (N)	3	1.2*	10851.1*	11.4*	0.04*	833.9*
T x N	3	0.4*	2574.9*	5.4*	0.01 ns	222.04 ns
Error		0.02	238.2	0.3	0.01	62.9

* significant at P≤0.05; ns - not significant.

Table S4. Correlation among metabolic profile of *A. flavus* and *A. flavus* aggressiveness under different pretreatments and treatments. Spearman's correlation coefficients (R) were calculated for metabolic profile of *A. flavus* and aggressiveness of *A. flavus* in infected pretreated FNP seeds, uninfected FNP seeds and control using STATISTICA 13.5.

	3-Nitropropionic acid	Aflatoxicol	Aflatoxin B1	Aflatoxin B2	Kojic acid	O-Methylsterigmatocystin	AUDPC standard
3-Nitropropionic a.		0.17	0.71	0.32	0.61	0.50	-0.35
Aflatoxicol	ns		0.42	0.04	0.68	0.30	0.14
Aflatoxin B1	***	*		0.58	0.85	0.76	-0.40
Aflatoxin B2	ns	ns	**		0.37	0.72	-0.30
Kojic acid	**	***	***	ns		0.71	-0.16
O-Methylsterigmatocystin	*	ns	***	***	***		-0.17
AUDPC standard	ns	ns	ns	ns	ns	ns	

*correlation is significant at the 0.05 level (2-tailed). **correlation is significant at the 0.01 level (2-tailed). ***correlation is significant at the 0.01 level (2-tailed). ns-not significant.

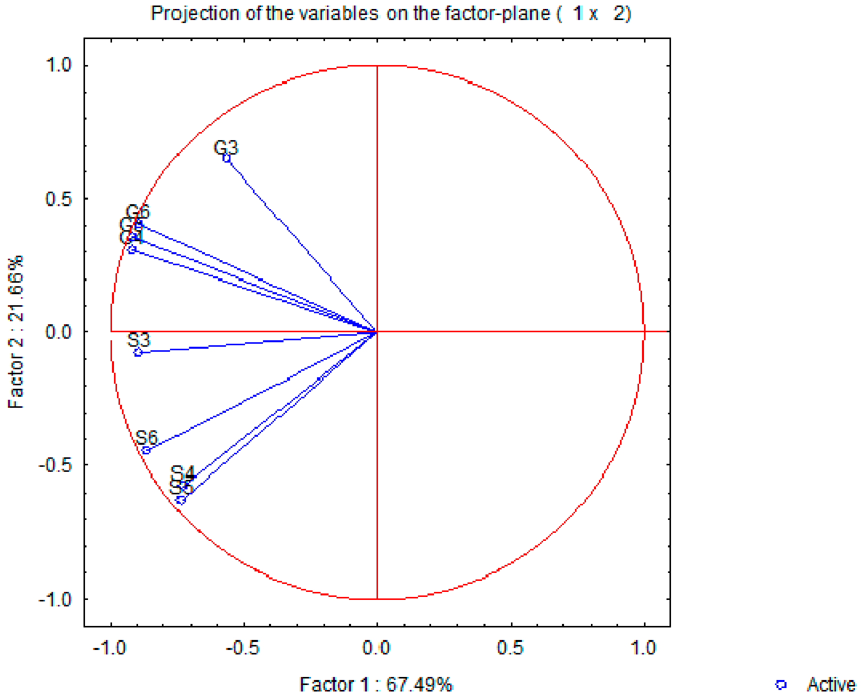

Figure S1. Principal component analysis of data sets of morphometric parameters under control (0), drought (PEG), nanoparticle (10, 100 and 1000 ng mL^{-1} FNP) and combined drought-nanoparticle treatments (10-PEG, 100-PEG and 1000-PEG). Loadings of first two factors. S3-S6 (shoot length from 3-6 days) and G3-G6 (germination from 3-6 days). PCA was used to distinguish the effect of single treatment application (control, drought or nanoparticles) and combined drought-nanoparticle treatment. Data were analysed by using STATISTICA 13.5 software package.
